# 10-HDA Induces ROS-Mediated Apoptosis in A549 Human Lung Cancer Cells by Regulating the MAPK, STAT3, NF-*κ*B, and TGF-*β*1 Signaling Pathways

**DOI:** 10.1155/2020/3042636

**Published:** 2020-12-08

**Authors:** Xin-Mei Lin, Shao-Bin Liu, Ying-Hua Luo, Wan-Ting Xu, Yu Zhang, Tong Zhang, Hui Xue, Wen-Bo Zuo, Yan-Nan Li, Bao-Xin Lu, Cheng-Hao Jin

**Affiliations:** ^1^Department of Food Science and Engineering, College of Food Science, Heilongjiang Bayi Agricultural University, Daqing 163319, China; ^2^Department of Biochemistry and Molecular Biology, College of Life Science & Technology, Heilongjiang Bayi Agricultural University, Daqing 163319, China; ^3^Department of Grass Science, College of Animal Science & Veterinary Medicine, Heilongjiang Bayi Agricultural University, Daqing 163319, China; ^4^National Coarse Cereals Engineering Research Center, Daqing 163319, China

## Abstract

10-Hydroxy-2-decenoic acid (10-HDA), also known as royal jelly acid, has a variety of physiological functions, and recent studies have shown that it also has anticancer effects. However, its anticancer mechanisms have not been clearly defined. In this study, we investigated the underlying mechanisms of 10-HDA in A549 human lung cancer cells. We used Cell Counting Kit-8 assay, scratch wound healing assay, flow cytometry, and western blot analysis to investigate its apoptotic effects and underlying mechanism. Our results showed that 10-HDA inhibited the proliferation of three types of human lung cancer cells and had no significant toxic effects on normal cells. Accompanying reactive oxygen species (ROS), 10-HDA induced A549 cell apoptosis by regulating mitochondrial-associated apoptosis, and caused cell cycle arrest at the G0/G1 phase in a time-dependent manner. Meanwhile, 10-HDA also regulated mitogen-activated protein kinase (MAPK), signal transducer and activator of transcription 3 (STAT3), and nuclear factor kappa B (NF-*κ*B) signaling pathways by increasing the expression levels of phosphorylated c-Jun N-terminal kinase, p-p38, and I-*κ*B, and additionally, by decreasing the expression levels of phosphorylated extracellular signal-regulated kinase, p-STAT3, and NF-*κ*B. These effects were blocked by MAPK inhibitors and *N*-acetyl-L-cysteine. Furthermore, 10-HDA inhibited cell migration by regulating transforming growth factor beta 1 (TGF-*β*1), SNAI1, GSK-3*β*, E-cadherin, N-cadherin, and vimentin. Taken together, the results of this study showed that 10-HDA induced cell cycle arrest and apoptosis in A549 human lung cancer cells through ROS-mediated MAPK, STAT3, NF-*κ*B, and TGF-*β*1 signaling pathways. Therefore, 10-HDA may be a potential therapy for human lung cancer.

## 1. Introduction

Lung cancer is a very serious illness, and its morbidity and mortality rank first among all cancer types [1]. In China, it ranks first in mortality, accounting for 24.41% of cancer-related deaths, and its mortality rate has shown an increasing trend [2]. The clinical manifestations of lung cancer are complex; the presence, severity, and appearance of symptoms and signs depend on the location of the tumor, the type of pathology, the presence or absence of metastases and complications, and the patient's response and tolerance [3]. At present, the main treatment methods for lung cancer include surgery, chemotherapy, radiation therapy, and molecular targeted therapy [4]. However, the mortality rate remains high, and these therapies often have side effects [5]. Therefore, it is urgent to find a natural anticancer drug that is safe and has high efficiency and low toxicity.

At present, the primary way for various active ingredients to exert anticancer activity is by triggering the apoptotic pathway in cancer cells, which leads to cell death [6–8]. Apoptosis is a programmed cell death mechanism controlled by genes and proteins, usually manifested as nuclear condensation, wrinkling, membrane foaming, and DNA fragmentation [9]. It is not a passive process, but is an active process that involves the activation, expression, and regulation of a series of intracellular proteins and complex signaling pathways [10–12]. Apoptosis occurs in the human body all the time, and the ultimate executor of apoptosis is caspase, which transmits signals from the point of origin to the various pathways of the cell, so that the cell reaches a globally consistent state of apoptosis, and finally decomposes the cell into small fragments [13]. Many studies have shown that some signaling pathways help promote cancer cell apoptosis, including MAPK, STAT3, and NF-*κ*B signaling pathways, and these pathways play key roles in cell apoptosis through the activation or inhibition of ROS [14, 15].

Recently, natural products have attracted the attention of many researchers for their potential anticancer properties [16]. 10-HDA, also known as royal jelly acid, is an organic acid compound extracted from royal jelly and is one of the main active ingredients [17, 18]. It has a variety of physiological functions such as antibacterial, anti-inflammatory, blood lipid lowering, immunity enhancing, and anticancer effects [19–24]. However, its pharmacodynamic effect and pharmacological mechanism of action in cancer remain unknown.

The main goal of this study was to reveal the target and pharmacological mechanism of 10-HDA in A549 lung cancer cells. Specifically, we measured its effects on cell viability, cell cycle, apoptosis, intracellular ROS production, inhibition of cell migration, potential molecular mechanisms, and related signaling pathways in lung cancer cells.

## 2. Materials and Methods

### 2.1. Chemicals and Reagents

10-HDA (Herbourify Co., Ltd., Chengdu, China) and 5-fluorouracil (5-FU; MedChemExpress, Princeton, NJ, USA) were dissolved in 100% dimethyl sulfoxide (Sigma-Aldrich, St. Louis, MO, USA) to obtain a 20 mM stock solution, and stored at -20°C before use. 2′,7′-Dichlorofluorescein diacetate (DCFH-DA; Merck Chemicals Shanghai Co., Ltd., Shanghai, China), an Apoptosis and Necrosis Assay Kit, an Annexin V-FITC Apoptosis Detection Kit, a Mitochondrial Membrane Potential Assay Kit with JC-1, and N-acetyl-L-cysteine (NAC) were purchased from Beyotime Institute of Biotechnology (Shanghai, China). The DNA Content Quantitation Assay (Cell Cycle) and Cell Counting Kit-8 (CCK-8) were purchased from Solarbio (Beijing, China). All of the antibodies were purchased from Santa Cruz Biotechnology, Inc. (Dallas, TX, USA). Other chemicals were of analytical grade.

### 2.2. Cell Lines and Cell Culture

Human lung cancer cell lines (A549, NCI-H460, and NCI-H23) and IMR90 human normal lung fibroblasts were purchased from the American Type Culture Collection (ATCC; Manassas, VA, USA). L-02 normal liver cells and GES-1 normal gastric cells were obtained from Saiqi Biological Engineering Co., Ltd. (Shanghai, China). A549, NCI-H460, and NCI-H23 cell lines were cultured in DMEM (Gibco, Waltham, MA, USA). IMR90, L-02, and GES-1 cell lines were cultured in RPMI-1640 (Gibco). All of the medium was supplemented with 10% heat-inactivated fetal bovine serum (Gibco), 100 U/mL penicillin, and 100 *μ*g/mL streptomycin (Gibco). The cells were maintained in a humidified atmosphere with 5% carbon dioxide at 37°C.

### 2.3. CCK-8 Assay

Cells were seeded onto cell slides in 96-well plates (1 × 10^4^ cells/well) for 24 h. Then, the cells were serum-starved in medium containing 1% FBS for 2 h. Then, the cells were treated with different concentrations (1, 3, 10, 30, and 100 *μ*M) of 5-FU (a common positive control of antitumor drugs) or 10-HDA for 24 h and treated with 30 *μ*M 5-FU or 10-HDA for different time periods (3, 6, 12, 24, and 36 h). At the indicated time points after transfection, 100 *μ*L culture medium containing 10% CCK-8 reagent (Solarbio) was added. The cells were subsequently incubated for 1 h at 37°C. Absorption intensity was analyzed using an automatic microplate reader (BioTek Instruments Inc., Winooski, VT, USA) at 450 nm. The half-maximal inhibitory concentration (IC_50_) was calculated using GraphPad Prism 5.0 software (GraphPad Software, Inc., San Diego, CA, USA).

### 2.4. Cell Apoptosis Analysis

A549 cells were seeded onto cell slides in 6-well plates (1 × 10^5^ cells/well) and treated with 30 *μ*M 10-HDA for different time periods (3, 6, 12, and 24 h). After washing twice with phosphate-buffered saline (PBS), cells were resuspended in 195 *μ*L binding buffer, and dual staining was performed with 3 *μ*L Hoechst 33342 and 2 *μ*L propidium iodide (PI). The staining solution was evenly distributed by shaking and incubated at 37°C for 3–5 min. The change in fluorescence intensity was observed using the EVOS FL Auto Cell Imaging System (Thermo Fisher Scientific, Waltham, MA, USA) at a magnification of 400x. Meanwhile, the effects of 10-HDA on the apoptosis of A549 cells were quantified using the Annexin V-FITC Apoptosis Detection Kit. A549 cells were treated with 30 *μ*M 10-HDA for different time periods (3, 6, 12, and 24 h). A549 cells were resuspended in 195 *μ*L Annexin V-FITC binding solution followed by the addition of 3 *μ*L Annexin V-FITC and 2 *μ*L PI and gentle mixing. Then, cells were incubated at 4°C for 30 min, and the cell suspension was transferred to a flow cytometer (Beckman Coulter, Brea, CA, USA) for quantitative apoptosis detection.

### 2.5. Analysis of the Mitochondrial Membrane Potential

A549 cells were plated in 6-well plates and treated with 30 *μ*M 10-HDA for different time periods (3, 6, 12, and 24 h), and collected in 10% DMEM. The cells were incubated with JC-1 working solution at 37°C for 20 min. Then, the supernatant was discarded, and the cells were washed twice with 1x JC-1 staining buffer solution. The cell suspension was transferred to a flow cytometer for detection of cellular MMP changes.

### 2.6. Analysis of ROS Generation

A549 cells were treated with 30 *μ*M 10-HDA for different time periods (3, 6, 12, and 24 h). The ROS inhibitor NAC was added 30 min before treatment with 10-HDA. Cells were centrifuged at 5,000 rpm for 5 min and washed twice with PBS. DCFH-DA was used to treat cells for 30 min at 37°C, and then washed twice with PBS. The cell suspension was transferred to a flow cytometer for detection of intracellular ROS levels.

### 2.7. Cell Cycle Analysis

A549 cells were treated with 30 *μ*M 10-HDA for different time periods (3, 6, 12, and 24 h). Cells were trypsinized and fixed in 70% ethanol for 12 h after washing with 1 mL PBS. Cell suspensions were incubated with 100 *μ*L RNase A and 400 *μ*L PI for 30 min without bright light at 37°C. The cell suspension was transferred to a flow cytometer for detection of cellular DNA content.

### 2.8. Cell Migration Analysis

A549 cells were seeded onto cell slides in 6-well plates (1 × 10^5^ cells/well). When the cells were completely fused, a wound was made by a 10 *μ*L pipette tip and washed twice with 1 mL PBS to remove cellular debris. Culture medium and 10-HDA (30 *μ*M) were added to continue the culture (3, 6, 12, and 24 h); then, the A549 cells were observed using the EVOS FL Auto Cell Imaging System at a magnification of 100x.

### 2.9. Western Blot Analysis

Collected cells were lysed using protein lysis solution. Equal amounts of proteins (30 *μ*g) were separated using 8–12% sodium dodecyl sulfate polyacrylamide gel electrophoresis and were subsequently transferred to nitrocellulose membranes (Millipore, Billerica, MA, USA). Then, the membranes were blocked in 5% skim milk for 2 h at 37°C. Next, the membranes were incubated for 12 h at 4°C with the following primary antibodies (all from Santa Cruz Biotechnology): mouse monoclonal antibodies against *α*-tubulin, B-cell lymphoma 2 (Bcl-2), Bcl-2-associated X protein (BAX), cleaved caspase-3 (caspase-3), cleaved poly(ADP ribose) ribose (PARP), cytochrome c (cyto-c), phosphorylated c-Jun N-terminal kinase (p-JNK), JNK, p-p38, p-extracellular signal-related kinase (p-ERK), p-STAT3, STAT3, NF-*κ*B (p65), inhibitor of NF-*κ*B alpha (I-*κ*B), TGF-*β*1, SNAI1, GSK-3*β*, E-cadherin, N-cadherin, and vimentin; and rabbit polyclonal antibodies against cyclin-dependent kinases 2/4/6 (CDK2/4/6), cyclin D1/E, p21, p27, ERK2, and p38*α*/*β*. Membranes were incubated with horseradish peroxidase-conjugated AffiniPure goat anti-mouse or goat anti-rabbit secondary antibodies (ZSGB-Bio, Inc., Beijing, China). Proteins were detected by enhanced chemiluminescence (Thermo Fisher Scientific) and imaged using the Amersham Imager 600 (GE Healthcare, Fairfield, CT, USA), and *α*-tubulin was used as the internal control.

### 2.10. Statistical Analysis

All experiments were repeated three times. The data were analyzed using SPSS 21.0 and expressed as the mean ± standard deviation (SD). Differences between groups were analyzed by one-way ANOVA. ^∗^*p* < 0.05, ^∗∗^*p* < 0.01, and ^∗∗∗^*p* < 0.001 indicated statistically significant differences.

## 3. Results

### 3.1. 10-HDA Inhibits the Proliferation of Human Lung Cancer Cells

**Figure 1:**
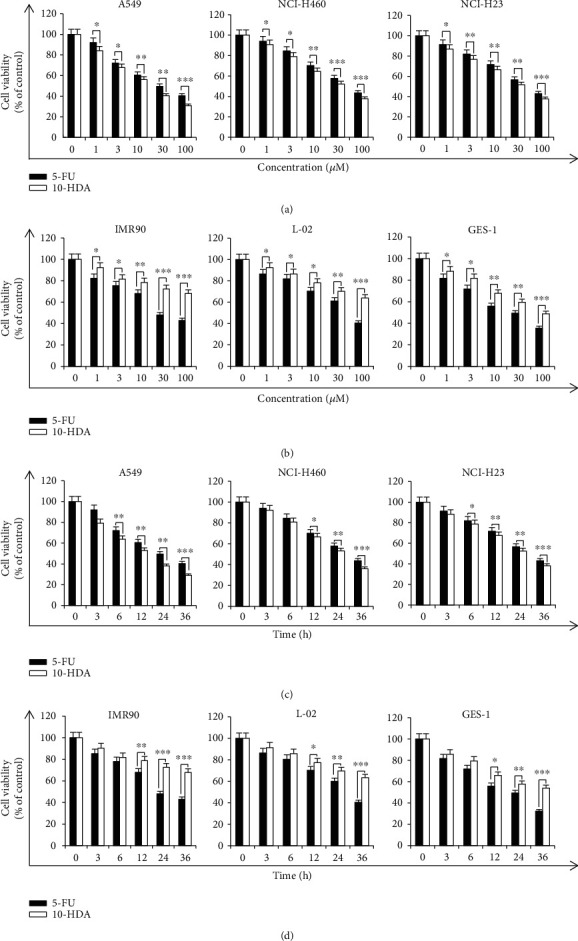
Effects of 10-HDA on the viability of human lung cancer cells. (a) A549, NCI-H460, and NCI-H23 human lung cancer cells were treated with different concentrations (1, 3, 10, 30, and 100 *μ*M) of 10-HDA and 5-FU for 24 h. (b) IMR90 human normal lung fibroblasts, L-02 human normal liver cells, and GES-1 human gastric epithelial mucosa cells were treated with different concentrations (1, 3, 10, 30, and 100 *μ*M) of 10-HDA and 5-FU for 24 h. (c) A549, NCI-H460, and NCI-H23 human lung cancer cells were treated with 30 *μ*M 10-HDA and 5-FU for different time periods (3, 6, 12, 24, and 36 h). (d) IMR90 human normal lung fibroblasts, L-02 human normal liver cells, and GES-1 human gastric epithelial mucosa cells were treated with 30 *μ*M 10-HDA and 5-FU for different time periods (3, 6, 12, 24, and 36 h). Cell viability was assessed by the CCK-8 assay. ^∗^*p* < 0.05, ^∗∗^*p* < 0.01, and ^∗∗∗^*p* < 0.001 vs. the 5-FU group.

As shown in Table 1 and Figures 1(a) and 1(c), 10-HDA inhibited the growth of all three human lung cancer cell lines in time- and concentration-dependent manners. Compared with the positive control 5-FU, the difference was statistically significant. The IC_50_ values were 44.72 *μ*M for 5-FU and 22.68 *μ*M for 10-HDA in A549 cells; 62.89 *μ*M for 5-FU and 44.03 *μ*M for 10-HDA in NCI-H460 cells; and 61.09 *μ*M for 5-FU and 44.79 *μ*M for 10-HDA in NCI-H23 cells. The A549 cell line was more sensitive to 10-HDA than the NCI-H460 and NCI-H23 cell lines. Meanwhile, we chose IMR90 human normal lung fibroblasts, L-02 normal liver cells, and GES-1 normal gastric cells as controls to directly reflect the toxic effects in the toxicity study. As shown in Figures 1(b) and 1(d), compared with 5-FU, the cytotoxic effects of 10-HDA on normal cells were less than those of 5-FU. These results indicated that 10-HDA has an excellent toxicity profile in human lung cancer cells.

### 3.2. 10-HDA Induces Apoptosis in A549 Human Lung Cancer Cells

**Figure 2:**
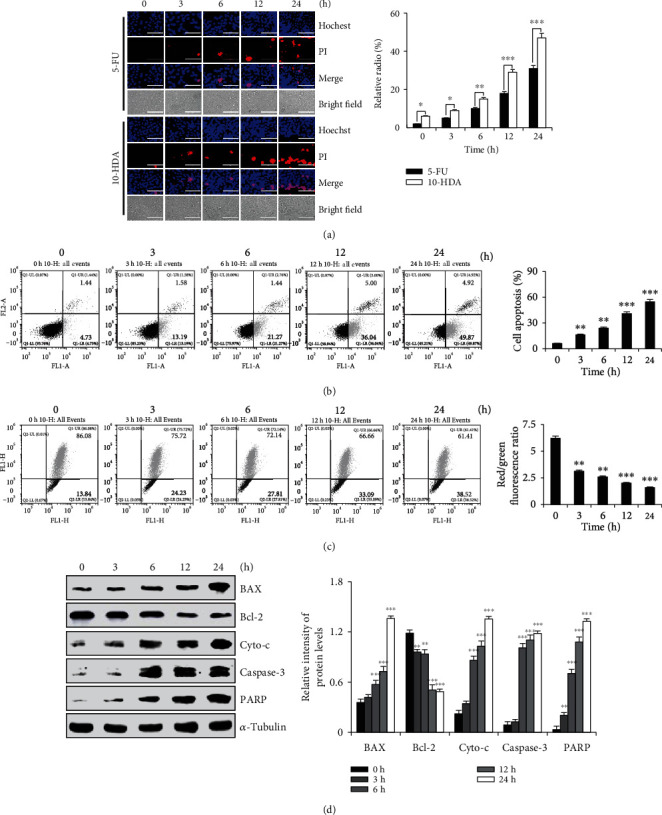
Effects of 10-HDA on the apoptosis of A549 human lung cancer cells. (a) A549 cells were treated with 30 *μ*M 10-HDA and 5-FU for different time periods (3, 6, 12, and 24 h), stained with Hoechst 33342 and PI, and observed by fluorescence microscopy. The scale bar is 100 *μ*m. (b) A549 cells were treated with 30 *μ*M 10-HDA for different time periods (3, 6, 12, and 24 h), and measured by flow cytometry. (c) A549 cells were treated with 30 *μ*M 10-HDA for different time periods (3, 6, 12, and 24 h), and MMP was detected by flow cytometry. (d) A549 cells were treated with 30 *μ*M 10-HDA for different time periods (3, 6, 12, 24, and 36 h), and the expression levels of apoptotic proteins (BAX, Bcl-2, cyto-c, caspase-3, and PARP) were detected by western blot analysis and were normalized to *α*-tubulin. The expression levels of proteins were analyzed by ImageJ software. ^∗^*p* < 0.05, ^∗∗^*p* < 0.01, and ^∗∗∗^*p* < 0.001 vs. the control group.

As shown in Figure 2(a), the cells became rounded, and the dead cells floated to the surface of the medium in the 10-HDA treatment groups. Meanwhile, as shown in Figure 2(b), the flow cytometry results showed that with an increase in 10-HDA treatment time, the number of early and late apoptotic cells increased to varying degrees, from 6.17% to 54.79%. In addition, when cell apoptosis occurred, the mitochondrial membrane potential (MMP) disappeared, membrane permeability changed, and a series of changes occurred. As shown in Figure 2(c), with increased treatment time, the fluorescence intensity of the cells continuously increased, and the proportion of depolarized cells was increased from 13.84% to 38.52%. As shown in Figure 2(d), the results indicated that 10-HDA treatment led to the downregulation of Bcl-2 and upregulation of BAX, cyto-c, caspase-3, and PARP in A549 cells. These results suggested that 10-HDA inhibits the proliferation of A549 human lung cancer cells through mitochondrial-dependent apoptosis.

### 3.3. 10-HDA Induces Apoptosis by Regulating MAPK, STAT3, and NF-*κ*B Signaling Pathways in A549 Human Lung Cancer Cells

**Figure 3:**
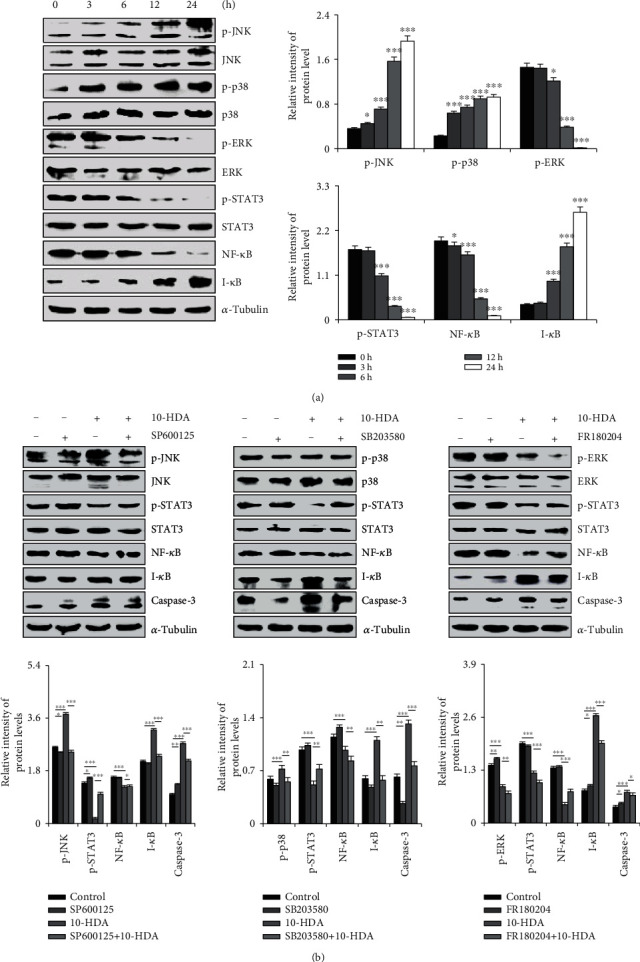
Effects of 10-HDA on MAPK, NF-*κ*B, and STAT3 signaling pathways in human lung cancer cells. (a) A549 cells were treated with 30 *μ*M 10-HDA for different time periods (3, 6, 12, 24, and 36 h), and then the expression levels of p38, JNK, ERK, NF-*κ*B, I-*κ*B, and STAT3 were detected by western blot analysis and were normalized to *α*-tubulin. (b) The cells were pretreated with 10 *μ*M of the JNK inhibitor (SP600125), p38 inhibitor (SB203580), and ERK inhibitor (FR180204) for 30 min, and then treated with 10-HDA for 24 h. The expression levels of p-JNK, p-p38, p-ERK, p-STAT3, NF-*κ*B, I-*κ*B, and caspase-3 were analyzed by western blotting and were normalized to *α*-tubulin. The expression levels of proteins were analyzed by ImageJ software. ^∗^*p* < 0.05, ^∗∗^*p* < 0.01, and ^∗∗∗^*p* < 0.001 vs. the control group and the NAC+10-HDA group.

As shown in Figure 3(a), the results indicated that 10-HDA treatment led to the upregulation of p-p38, p-JNK, and I-*κ*B and the downregulation of p-ERK, p-STAT3, and NF-*κ*B in A549 cells. As shown in Figure 3(b), when a JNK inhibitor (SP600125) and a p38 inhibitor (SB203580) were added, the inhibitory effect of 10-HDA was alleviated and p-STAT3 and NF-*κ*B were upregulated. There was a limited activation effect of 10-HDA and a limited downregulation of p-JNK, p-p38, I-*κ*B, and caspase-3. The ERK inhibitor (FR180204) enhanced the inhibitory effect of 10-HDA and the downregulation of p-ERK, p-STAT3, and NF-*κ*B, but it enhanced the upregulation of I-*κ*B. It also increased the activation ability of 10-HDA and the upregulation of caspase-3. These data suggested that 10-HDA induced apoptosis in A549 human lung cancer cells through regulating the MAPK, NF-*κ*B, and STAT3 signaling pathways.

### 3.4. 10-HDA Induces Apoptosis by Regulating Intracellular ROS Generation in A549 Human Lung Cancer Cells

**Figure 4:**
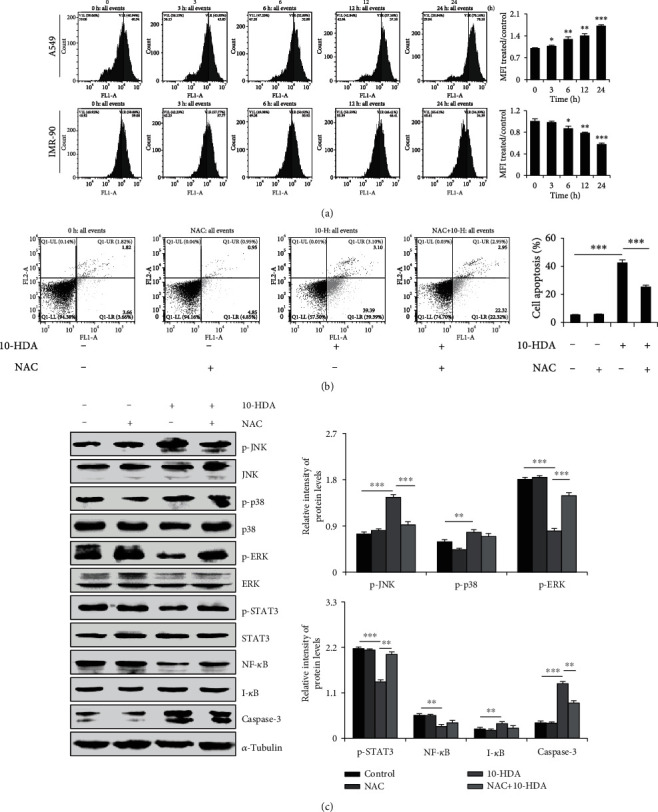
Effects of ROS generation and apoptosis after treatment of human lung cancer cells with 10-HDA. (a) A549 cells and IMR90 human normal lung fibroblasts were treated with 30 *μ*M 10-HDA after 24 h. ROS levels were examined by flow cytometry. (b) A549 cells were cultured with 30 *μ*M 10-HDA or 0.25 *μ*M (20 *μ*L/mL) NAC for 24 h, and cell apoptosis was detected by flow cytometry analysis. (c) A549 cells were pretreated with 0.25 *μ*M (20 *μ*L/mL) and NAC for 30 min, followed by treatment with 10-HDA for 24 h. The expression levels of MAPK, STAT3, NF-*κ*B, and caspase-3 were detected by western blot analysis and normalized to *α*-tubulin. The expression levels of proteins were analyzed with ImageJ software. ^∗^*p* < 0.05, ^∗∗^*p* < 0.01, and ^∗∗∗^*p* < 0.001 vs. the control group and the NAC+10-HDA group.

As shown in Figure 4(a), with 10-HDA treatment, intracellular ROS levels in the human lung cancer cells were significantly increased from 40.94% to 70.16% in a time-dependent manner, and intracellular ROS levels in IMR90 human normal lung cancer cells were significantly decreased from 59.08% to 34.39% in a time-dependent manner. As shown in Figure 4(b), after incubation with 10-HDA+NAC, compared with 10-HDA treatment alone, the number of apoptotic cells was significantly reduced from 42.49% to 25.27%. Meanwhile, as shown in Figure 4(c), compared with the control group, 10-HDA significantly led to the upregulation of p-p38, p-JNK, I-*κ*B, and caspase-3 in a time-dependent manner, and it also led to the downregulation of p-ERK, p-STAT3, and NF-*κ*B. NAC treatment alone also showed no significant changes compared to the control group. However, after incubation with 10-HDA+NAC, compared with 10-HDA treatment alone, scavenging of ROS by NAC significantly blocked MAPK, STAT3, and NF-*κ*B signaling pathways and decreased the caspase-3 levels. These results suggested that 10-HDA increased the levels of ROS in A549 cells, leading to apoptosis.

### 3.5. 10-HDA Triggers G0/G1 Phase Cell Cycle Arrest in A549 Human Lung Cancer Cells

**Figure 5:**
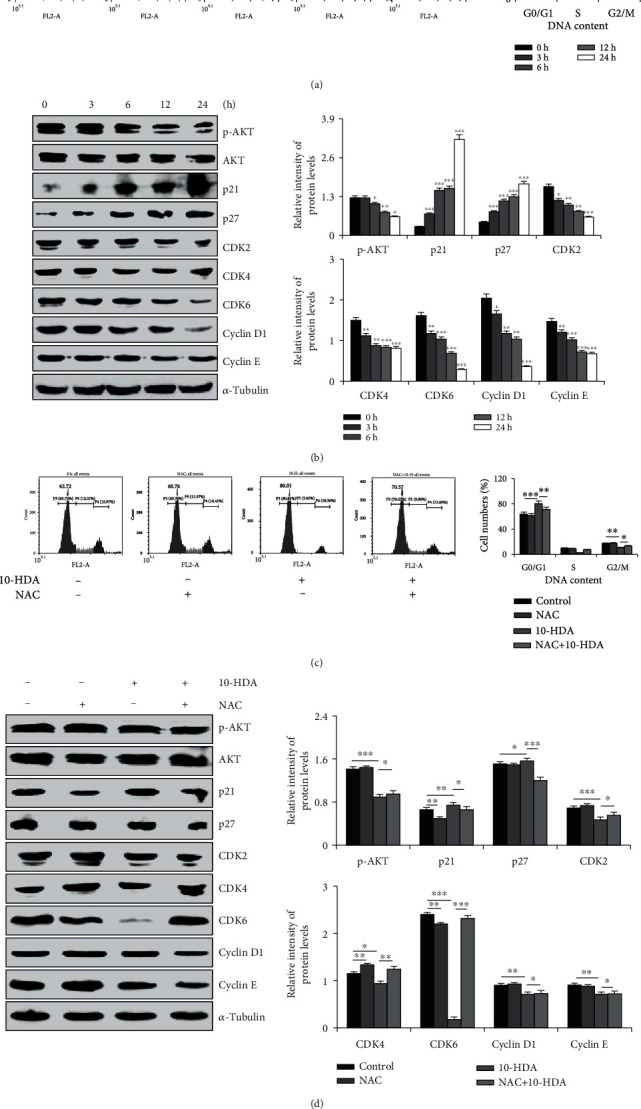
Effects of 10-HDA on cell cycle distribution and cell cycle checkpoint-related proteins of human lung cancer cells. (a) The cells were treated with 10-HDA for different time periods (3, 6, 12, and 24 h). A549 cells were stained with 100 *μ*L RNase A and 400 *μ*L PI; DNA content was analyzed for cell cycle phase distribution by flow cytometry. (b) Western blotting with antibodies against p-AKT, AKT, p21, p27, CDK2/4/6, and cyclin D1/E was normalized to *α*-tubulin. (c) A549 cells were cultured with 30 *μ*M 10-HDA or 0.25 *μ*M (20 *μ*L/mL) NAC for 24 h and detected by flow cytometric analysis. (d) A549 cells were pretreated with 0.25 *μ*M (20 *μ*L/mL) and NAC for 30 min, followed by treatment with 10-HDA for 24 h. The expression levels of p-AKT, AKT, p21, p27, CDK2/4/6, and cyclin D1/E were normalized to *α*-tubulin. The expression levels of proteins were analyzed by ImageJ software. ^∗^*p* < 0.05, ^∗∗^*p* < 0.01, and ^∗∗∗^*p* < 0.001 vs. the control group and the NAC+10-HDA group.

As shown in Figure 5(a), with increased 10-HDA treatment time, the percentage of cells in the G0/G1 phase increased over time, from 62.97% to 80.54%. As shown in Figure 5(b), 10-HDA treatment led to the downregulation of AKT, CDK2/4/6, and cyclin D1/E, and it also led to the upregulation of p21 and p27 in A549 cells. As shown in Figure 5(c), upon treatment with 10-HDA alone, compared to incubation with 10-HDA+NAC, the percentage of cells in the G0/G1 phase decreased, from 80.01% to 70.57%. As shown in Figure 5(d), compared with the control group, 10-HDA significantly led to the downregulation of p-AKT, CDK2/4/6, and cyclin D1/E, and it also led to the upregulation of p21 and p27. NAC treatment alone also showed no significant changes compared to the control group. After incubation with 10-HDA+NAC, compared with 10-HDA treatment alone, 10-HDA+NAC induced increased expression of p-AKT, CDK2/4/6, and cyclin D1/E, and it also induced decreased expression of p21 and p27. These results demonstrated that 10-HDA induced cell cycle arrest by regulating cell cycle-associated protein expression in A549 cells.

### 3.6. 10-HDA Inhibits Cell Migration by Regulating TGF-*β*1 Signaling Pathways in Human Lung Cancer A549 Cells

**Figure 6:**
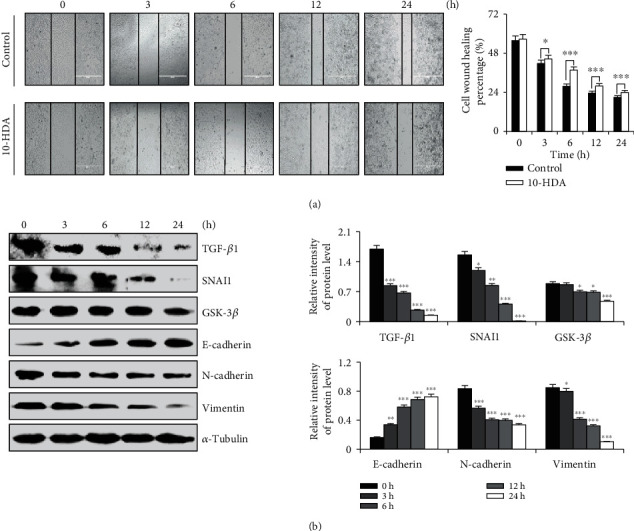
Effects of cell migration after treatment of human lung cancer cells with 10-HDA. (a) A549 cells were treated 3, 6, 12, and 24 h with 10-HDA, using the EVOS FL Auto Cell Imaging System at a magnification of 100x to observe. (b) A549 cells were treated with 10-HDA for 3, 6, 12, and 24 h to detect the levels of apoptotic proteins (TGF-*β*1, SNAI1, GSK-3*β*, E-cadherin, N-cadherin, and vimentin) and were normalized to *α*-tubulin. The expression levels of proteins were analyzed by ImageJ software. ^∗^*p* < 0.05, ^∗∗^*p* < 0.01, and ^∗∗∗^*p* < 0.001 vs. the control group.

As shown in Figure 6(a), compared with the control group, the human lung cancer cell intracellular migration was inhibited obviously under 10-HDA treatment in a time-dependent manner. As shown in Figure 6(b), the expression levels of TGF-*β*1, SNAI1, GSK-3*β*, N-cadherin, and vimentin were decreased, and the expression level of E-cadherin was also increased. These results indicated that 10-HDA can inhibit the migration of human lung cancer cells.

## 4. Discussion

10-HDA has garnered wide attention in recent years because of its various biological and pharmacological activities. In this study, we investigated the effects of 10-HDA on inhibiting cell proliferation, cell cycle arrest, and the induction of apoptosis in lung cancer cells. We evaluated the cytotoxic effects of 10-HDA on human lung cancer A549, NCI-H460, and NCI-H23 cells and found that 10-HDA significantly inhibited the proliferation of A549, NCI-H460, and NCI-H23 cells and had little cytotoxicity in normal IMR90, L-02, and GES-1 cells.

Apoptosis is process of programmed cell death with spontaneous characteristics. There are two ways to activate apoptosis: through intrinsic and extrinsic pathways [25]. Millions of cells in the human body undergo programmed cell death every hour; at the same time, millions of new proliferating cells replace these apoptotic cells, allowing tissues and organs to maintain their physiological functions for a long time. During the apoptotic process, it is mediated by the antiapoptotic protein Bcl-2 and proapoptotic protein BAX, which increases membrane permeability. Cyto-c is released into the cytosol and subsequently participates in the process leading to caspase-9 and caspase-3 activation [26]. Our results showed that after 10-HDA treatment of A549 human lung cancer cells, the expression level of antiapoptotic protein Bcl-2 decreased, and the expression of proapoptotic proteins BAX, cyto-c, caspase-3, and PARP increased in a time-dependent manner. These results suggest that 10-HDA can regulate the expression of Bcl-2 and BAX, and induce caspase-3-dependent apoptosis via the mitochondrial pathway.

Studies have reported that there is a wide range of interaction mechanisms between the MAPK STAT3 and NF-*κ*B signaling pathways [27, 28]. The MAPK family involves three major subgroups including ERK1/2, JNK, and p38 kinase. ERK1/2 is activated primarily by mitogenic stimuli such as growth factors leading to cell growth and survival [29, 30]. Here, we showed that 10-HDA increased the phosphorylation of p38 and JNK, and decreased the phosphorylation of ERK in A549 cells in a time-dependent manner. These results confirm that 10-HDA activates the p38 and JNK signaling pathways through protein phosphorylation, and inhibits the ERK signaling pathway, further inhibiting STAT3 and NF-*κ*B activity, resulting in cell apoptosis.

ROS, as a natural by-product of aerobic respiration, is closely related to cell apoptosis, cell cycle, signal transduction cascade, protein phosphorylation, and cytoskeleton formation [31, 32]. Increased ROS stimulates the cancer-related signal transduction pathway and enhances the survival and proliferation of cancer cells [33]. ROS can also be used as a signaling molecule to transduce extracellular stimulus signals, directly inducing apoptosis or indirectly participating in intracellular signal transduction [34]. In this study, 10-HDA induced the generation of ROS in A549 cells and inhibited the generation of ROS in normal cells, both in a time-dependent manner. However, when the cells were treated with NAC and 10-HDA, NAC had no effect on the control group, but had a strong inhibitory effect on the 10-HDA group. Thus, 10-HDA stimulated ROS after entering the cells. It mediated the pathways of MAPK, STAT3, and NF-*κ*B to inhibit A549 cell proliferation and control the growth and metastasis of cells, thereby achieving anticancer effects.

The uncontrolled proliferation of cancer cells is closely related to the regulatory mechanism of cell cycle progression, and it is also a significant feature of accelerating tumor growth [35]. In the present study, we demonstrated that 10-HDA induced G0/G1 cell cycle arrest in A549 cells by flow cytometry, as well as the molecular mechanisms underlying the regulation of the cell cycle processes. The cyclin D1 gene, which is tightly correlated with cancerous cell proliferation, has been considered a marker molecule [36]. Similarly, cyclin E and CDK2/4/6 also play key roles in the G0/G1 transition in the cell cycle [37–40]. Furthermore, p21 and p27 act by binding CDK in the G1 phase of the cell cycle, leading to inhibition of the phosphorylation of other proteins such as retinoblastoma, which is necessary for cell cycle progression [41, 42]. In our study, protein analysis showed the decreased expression of cyclin D1/E and CDK2/4/6, and increased expression of p21 and p27 proteins. However, 10-HDA cotreatment with NAC led to the decrease in Akt. These results suggest that ROS generation can inhibit the phosphorylation of Akt, thereby activating formation of the CDK2/4/6 and cyclin D1/E kinase complex [43]. These results indicated that 10-HDA triggered cell cycle arrest at the G0/G1 phase in A549 human lung cancer cells by downregulating the expression of Akt, CDK2/4/6, and cyclin D1/E and also by upregulating p21 and p27.

It is well known that inhibiting cancer cell migration has important significance for the treatment of cancer. The TGF-*β*1 signaling pathway has been confirmed to modulate numerous physiologic processes, including proliferation, migration, and invasion of tumors [44, 45]. Activation of TGF-*β*1 signaling may affect the crucial role in cells through activating downstream factors (GSK-3*β*) [46, 47]. Furthermore, TGF-*β*1 signaling was the most enriched pathway by ectopic expression of Akt and MAPK pathways, all of which were engaged in cell proliferation and migration, and some studies also found that the inhibition of cell migration induced by the TGF-*β*1 signaling pathway presumably attributed to the suppression of ROS-dependent mechanisms [48, 49]. In our study, we found that the expression level of E-cadherin was upregulated, and those of TGF-*β*1, SANI 1, GSK-3*β*, N-cadherin, and vimentin were decreased to different extents. These results showed that 10-HDA regulated the signal transduction pathways in A549 cells, so that the cells could not proliferate normally and limiting their range of activities, thereby inhibiting the migration of lung cancer cells.

In the present study, we demonstrated that 10-HDA induces ROS-mediated apoptosis in A549 human lung cancer cells by regulating the MAPK, STAT3, NF-*κ*B, and TGF-*β*1 signaling pathways. The effects of 10-HDA demonstrated in vivo should be evaluated in future studies.

## 5. Conclusion

**Figure 7:**
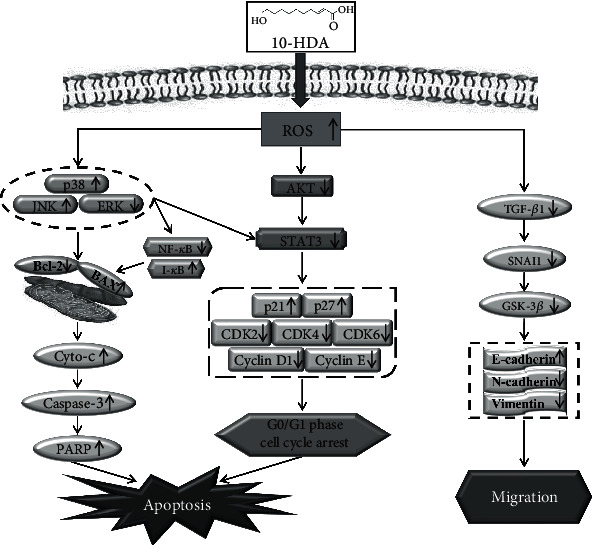
Mechanism of 10-HDA in A549 human lung cancer cells.

In conclusion, 10-HDA induced apoptosis and cell cycle arrest of A549 human lung cancer cells through ROS-mediated modulation of the MAPK, STAT3, NF-*κ*B, and TGF-*β*1 signaling pathways, and it also induced eventual mitochondrial-dependent apoptosis (Figure 7). These findings indicate that 10-HDA may be a potential therapy for human lung cancer.

## Figures and Tables

**Table 1:** IC_50_ values of 10-HDA in lung cancer cells.

Cell name	5-FU (*μ*M)	10-HDA (*μ*M)
A549	44.72 ± 2.01	22.68 ± 1.08
NCI-H460	62.89 ± 2.33	44.03 ± 1.52
NCI-H23	61.09 ± 1.89	44.79 ± 1.77

## Data Availability

The data used to support the findings of this study are available from the corresponding authors upon request.
